# Agro-Climatic Variation in Wheat Growth, Yield, and Grain Quality Across South Korea

**DOI:** 10.3390/plants15182852

**Published:** 2026-09-18

**Authors:** Hyun Hwa Park, Pyae Pyae Win, Yong In Kuk

**Affiliations:** Department of Bio-Oriental Medicine Resources, Sunchon National University, Suncheon 57922, Republic of Korea; camelia9720@scnu.ac.kr (H.H.P.); winpyae581@gmail.com (P.P.W.)

**Keywords:** agro-climatic regions, cultivation suitability, grain quality, soil fertility, wheat, winter temperature

## Abstract

Wheat cultivation has recently expanded into new agro-climatic regions of South Korea owing to increasing concerns about food security and changing climatic conditions. However, comprehensive evaluations of regional cultivation suitability remain limited. This study evaluated regional variations in wheat growth, yield, grain quality, and cultivation suitability across three agro-climatic groups classified primarily by January minimum temperature: G1 (<−10 °C; central inland and high-altitude regions), G2 (−8 to −7 °C; southern inland and plain regions), and G3 (−4 to −3 °C; southern coastal and warmer regions). During the 2024 and 2025 growing seasons, field experiments were conducted, and growth traits, yield components, meteorological data, soil properties, and grain quality were analyzed. Principal component analysis (PCA) was used to examine the relationships among agronomic and grain quality traits. In both years, G3 exhibited superior growth performance, characterized by earlier heading, higher biomass, and greater tiller and spike numbers, resulting in the highest grain yield (4.3 t ha^−1^), compared with G1 (2.8 t ha^−1^). The superior growth and yield performance observed in G3 was associated with relatively higher winter temperatures and differences in soil nutrient statuses, although other environmental factors may also have contributed. In contrast, the lower productivity observed in G1 may have been related to colder winter conditions and differences in soil nutrient statuses. G2 demonstrated a balance between productivity and stability. Grain quality traits also exhibited regional variability. PCA further indicated that G3 was associated with productivity-related traits, whereas G1 was associated with crude protein content and grain quality-related characteristics. Overall, regional differences in winter temperature and soil fertility were associated with contrasting patterns of wheat productivity and grain quality. These findings highlight the importance of integrated, region-specific cultivation strategies for optimizing wheat production under changing climatic conditions.

## 1. Introduction

Wheat (*Triticum aestivum* L.), a staple food worldwide, has received increasing attention in South Korea in relation to food security and self-sufficiency. Recently, instability in global grain markets, increasing dependence on imported grain supplies, and more frequent climate-related extreme weather events have revealed structural vulnerabilities in national food supply systems [1,2,3]. As a result, increasing the domestic wheat supply and establishing stable systems for local cultivation have become matters of national interest.

Therefore, the Korean government has announced several policies, including increasing certified seed production, supporting research on breeding localized wheat varieties, and promoting mechanized cultivation [2]. Certified seeds have also been distributed to nearly all administrative regions in the country, and wheat management has been shifting toward a national production system since 2024. Wheat cultivation has recently expanded in central inland and northern regions, even at high altitudes, where cultivation was previously less prevalent. This expansion may be partly associated with rising winter temperatures, which can improve overwintering survival and potentially expand the northern cultivation boundary [4,5,6]. In South Korea, the minimum temperature in January has traditionally been regarded as one of the most important climatic indicators for determining the northern cultivation boundary of winter wheat. Regions with average minimum temperatures below approximately −10 °C are generally considered marginal for stable overwintering and wheat production [7]. Consequently, agro-climatic zones are often classified into northern inland/high-altitude, intermediate inland, and southern coastal production regions based on winter temperature conditions. However, it remains unclear whether this traditional temperature threshold adequately reflects current regional differences in wheat growth, yield, grain quality, and cultivation suitability under recent climatic conditions, as comprehensive field-based evaluations across agro-climatic regions remain limited.

Recent increases in winter temperatures are expected to shift the cultivation boundary of winter wheat northward and into higher-altitude regions in South Korea. Consequently, evaluating wheat performance across diverse agro-climatic regions is essential to determine whether these newly expanded cultivation areas can sustain stable growth, yield, and grain quality comparable to those of traditional wheat-producing regions and to assess future cultivation suitability under changing climatic conditions. Previous field-based studies in South Korea have shown that regional temperature variation influences crop growth, yield, and grain quality in barley [7]. In particular, minimum winter temperatures, diurnal temperature variation, and soil nutrient availability, including nitrogen and potassium, are critical factors affecting tillering, heading, and grain-filling processes [6,8,9]. These environmental factors may ultimately influence yield stability and grain quality.

In addition to yield performance, grain quality, particularly its nutritional and functional attributes, has become increasingly important in modern wheat production. Consequently, increasing attention has been paid to bioactive compounds, such as crude protein, phenolics, flavonoids, and glutathione (GSH), which contribute to the nutritional value, antioxidant capacity, and industrial utilization of wheat grain [10,11,12]. Therefore, understanding how regional environmental conditions influence both productivity and functional grain quality is essential for developing climate-resilient and high-value wheat production systems. As regional cultivation suitability is determined by the combined effects of climatic, soil, agronomic, and grain quality traits, principal component analysis (PCA) provides a useful approach for integrating multiple variables and identifying the major factors associated with regional adaptation and cultivation suitability [13].

Accordingly, this study was conducted to evaluate regional differences in wheat growth, yield, grain quality, and environmental conditions across the major wheat-growing areas of South Korea. We hypothesized that regions with relatively warmer winter temperatures and higher soil fertility would exhibit improved wheat growth and yield performance, accompanied by differences in grain quality, resulting in distinct cultivation suitability among agro-climatic zones. Therefore, the objectives of this study were to (i) compare growth, yield, and grain quality traits among agro-climatic regions; (ii) evaluate the relationships between climatic and soil factors and wheat performance; and (iii) evaluate regional wheat production performance and its implications for management under changing climatic conditions. This integrative approach provides new insights into the relationships between environmental conditions and wheat performance and offers a scientific basis for region-specific wheat cultivation under changing climatic conditions.

## 2. Results

### 2.1. Growth Characteristics at Early and Heading Stages by Region

**Figure 1:**
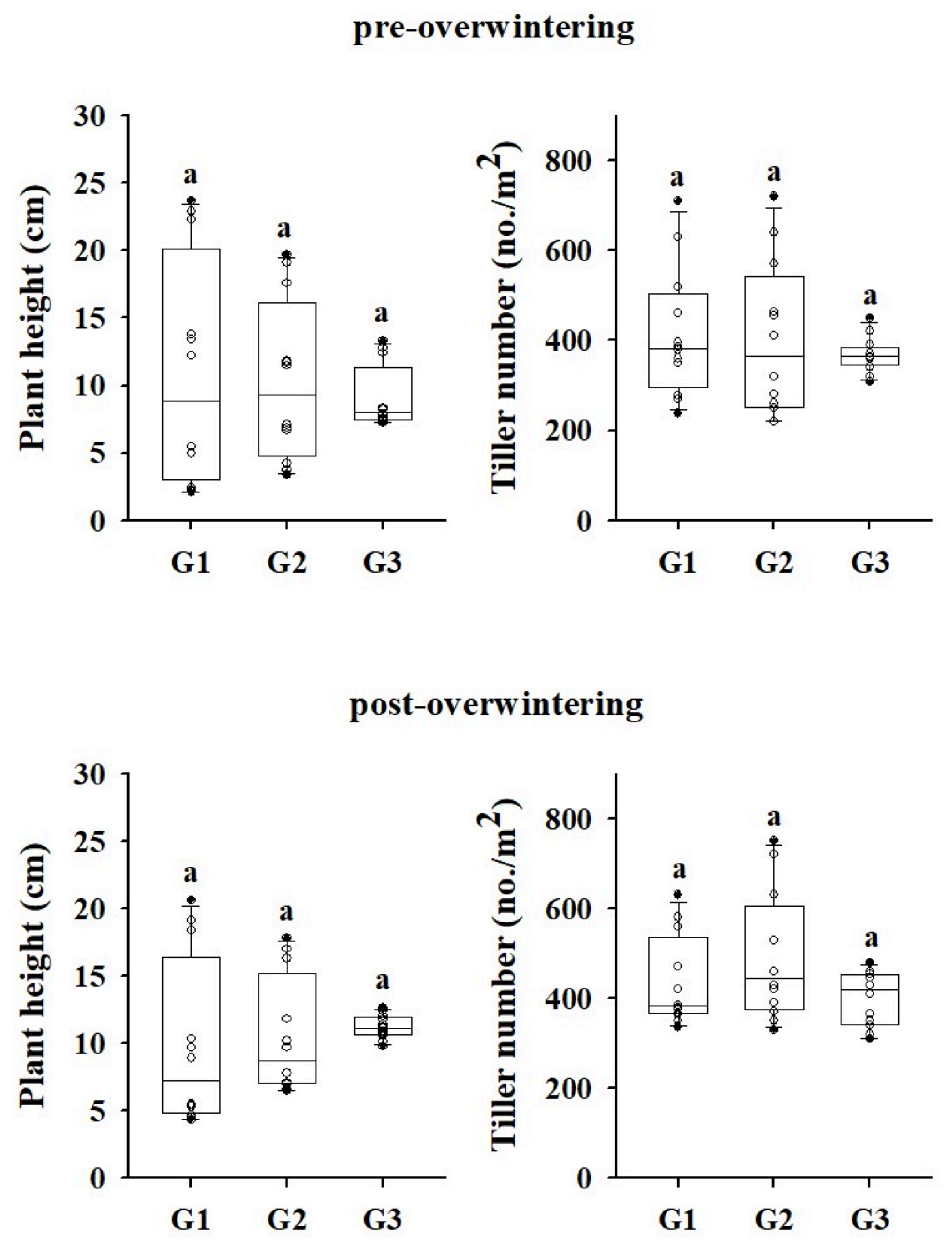
Growth of wheat measured at pre– overwintering and post–overwintering growth stages among regional groups (G1–G3) in 2025. Boxplots show interquartile ranges with the bold line indicating the median; points represent individual observations. Individual points represent replicate sampling plots (*n* = 12 per agro-climatic group). Different letters indicate significant differences among agro-climatic groups within each growth stage based on estimated marginal means with Tukey adjustment (*p* < 0.05). Detailed statistical results are provided in Table S1.

**Figure 2:**
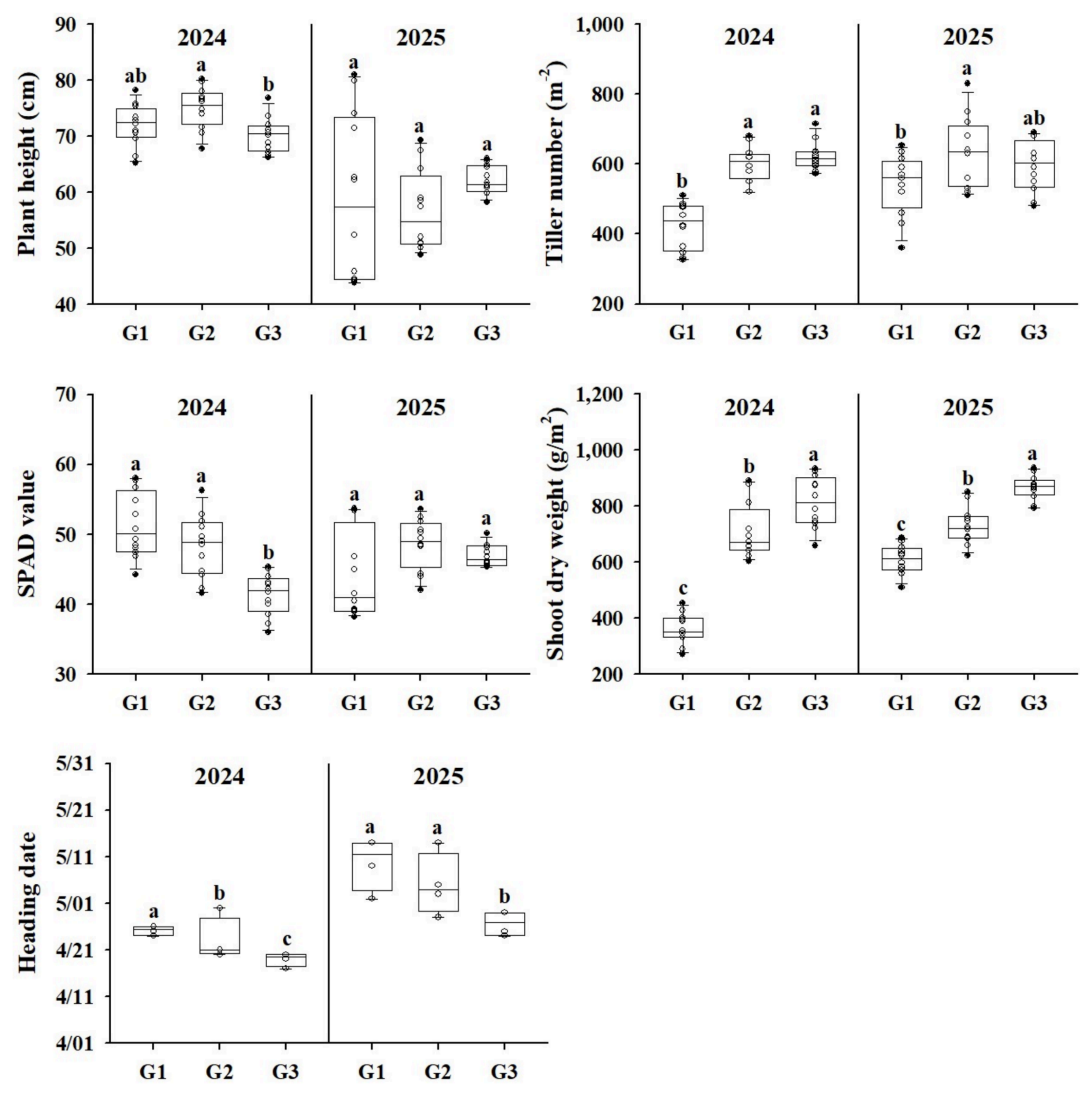
Growth and chlorophyll content (SPAD) of wheat at the heading growth stage among regional groups (G1–G3) in 2024 (**left**) and 2025 (**right**). Boxplots are presented as described in Figure 2. Individual points represent replicate sampling plots (*n* = 12 per agro-climatic group in each year). Different letters indicate significant differences among regional groups within each year based on estimated marginal means with Tukey adjustment (*p* < 0.05). Effects of group, year, and the Group × Year interaction were evaluated using linear mixed-effects models, with detailed statistical results provided in Table S2.

Pre- and post-regeneration growth evaluations were conducted in 2025, whereas all measurements from the heading stage onward were performed in both 2024 and 2025. During the pre-wintering period in December, plant height was comparable between G1 and G2 and slightly lower in G3; however, these differences were not statistically significant. Similarly, no significant regional differences were observed in the number of tillers. After regeneration in February, plant height was higher in G3 than in G1 and G2, although the differences were not significant (Figure 1; Table S1). Plant height at the heading stage showed generally similar patterns among regions in 2024 and 2025 (Figure 2). Although the overall group effect on plant height was not significant, significant effects of year and the Group × Year interaction were detected (Table S2). The number of tillers was generally lower in G1, with significant effects of group, year, and the Group × Year interaction. Chlorophyll content was significantly lower in G3 than in G1 and G2 in 2024, whereas no significant regional differences were observed in 2025. The overall group and year effects on SPAD values were not significant, but the Group × Year interaction was significant. Shoot dry weight generally increased in the order G1 < G2 < G3, with significant effects of group, year, and the Group × Year interaction. Heading date also differed significantly by group and year, with a significant Group × Year interaction. In 2024, heading occurred in the order G3 < G2 < G1, whereas in 2025, heading in G3 was significantly earlier than in G1 and G2. Overall, G3 tended to show earlier heading and greater shoot biomass than G1, although the magnitude of regional differences varied between years.

### 2.2. Growth and Yield Components at Harvest by Region

**Figure 3:**
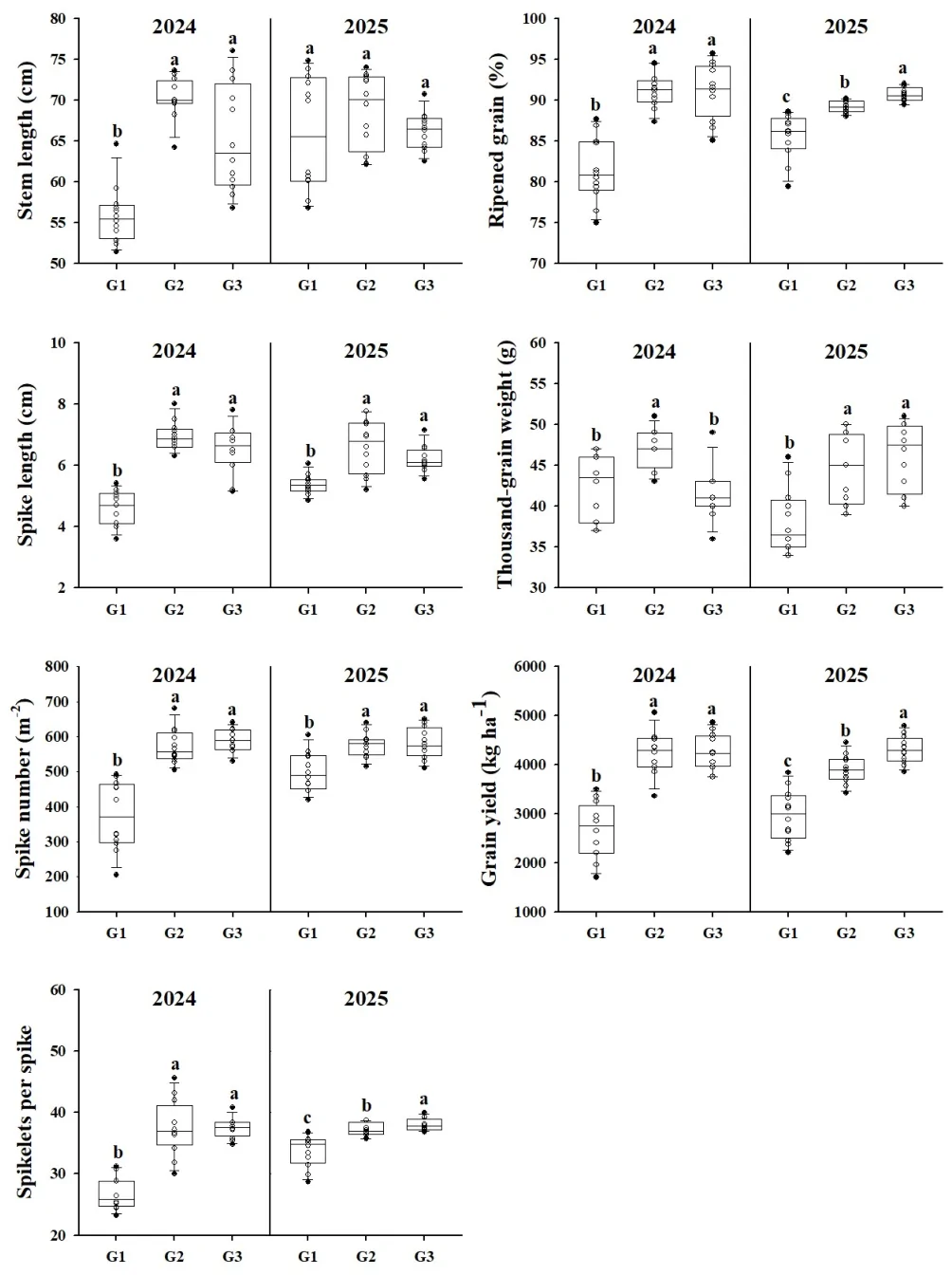
Growth and yield components of wheat at the harvest stage among regional groups (G1–G3) in 2024 (**left**) and 2025 (**right**). Boxplots are presented as described in Figure 2. Individual points represent replicate sampling plots (*n* = 12 per agro-climatic group in each year). Different letters indicate significant differences among agro-climatic groups within each year based on estimated marginal means with Tukey adjustment (*p* < 0.05). Effects of group, year, and the Group × Year interaction were evaluated using linear mixed-effects models, with detailed statistical results provided in Table S3.

A comparison of growth and yield components among the agro-climatic groups (G1–G3) in 2024 and 2025 showed that most traits tended to be higher in G2 and G3 than in G1 (Figure 3). The mixed-effects analysis revealed significant Group × Year interactions for all harvest-stage growth and yield-related traits (Table S3), indicating that the magnitude of regional differences varied between years. Stem length was generally greater in G2 and G3 than in G1, with significant effects of group, year, and the Group × Year interaction. Spike length, spike number, spikelets per spike, and ripened grain percentage were generally lower in G1 than in G2 and G3, although the magnitude of these regional differences varied between years. Thousand-grain weight showed no significant overall group or year effect, but a significant Group × Year interaction was detected. Grain yield was significantly higher in G2 and G3 than in G1 in 2024, whereas significant differences were observed among all three groups in 2025, in the order G3 > G2 > G1. Grain yield showed a significant group effect and Group × Year interaction, whereas the overall year effect was not significant.

### 2.3. PCA Based on Growth and Yield Components at Harvest

**Figure 4:**
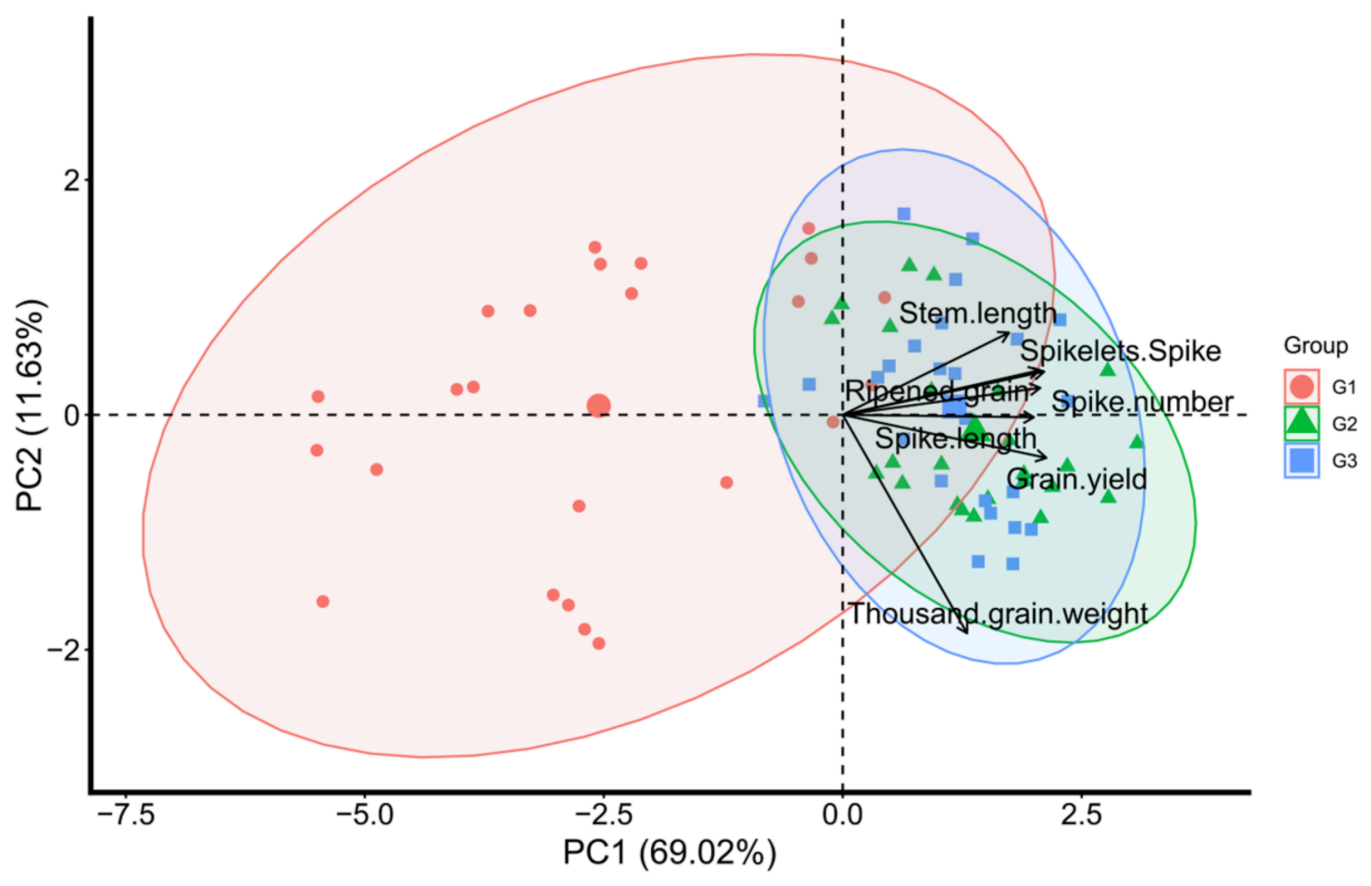
Principal component analysis biplot of growth and yield components of wheat at the harvest stage by regional groups (G1–G3) in 2024 and 2025. The analysis included 24 plot-level observations per agro-climatic group (*n* = 72 in total). Eigenvalues, explained variance, variable loadings, and contributions are provided in Table S4.

PCA was conducted using seven harvest-stage growth and yield-related traits: stem length, spike length, spike number, spikelets per spike, ripened grain percentage, thousand-grain weight, and grain yield (Figure 4; Table S4). The first two principal components explained 80.6% of the total variation, with PC1 and PC2 accounting for 69.0% and 11.6%, respectively. PC1 was mainly associated with spikelets per spike, spike number, grain yield, spike length, and ripened grain percentage, whereas PC2 was primarily associated with stem length and thousand-grain weight. The PCA biplot showed differentiation among the three agro-climatic groups along the first two principal components. G3 was positioned closer to several yield-related traits, whereas G2 showed a broadly similar pattern. Overall, the PCA indicated regional differentiation in harvest-stage growth and yield-related traits.

### 2.4. Regional Weather and Soil Environmental Factors

**Figure 5:**
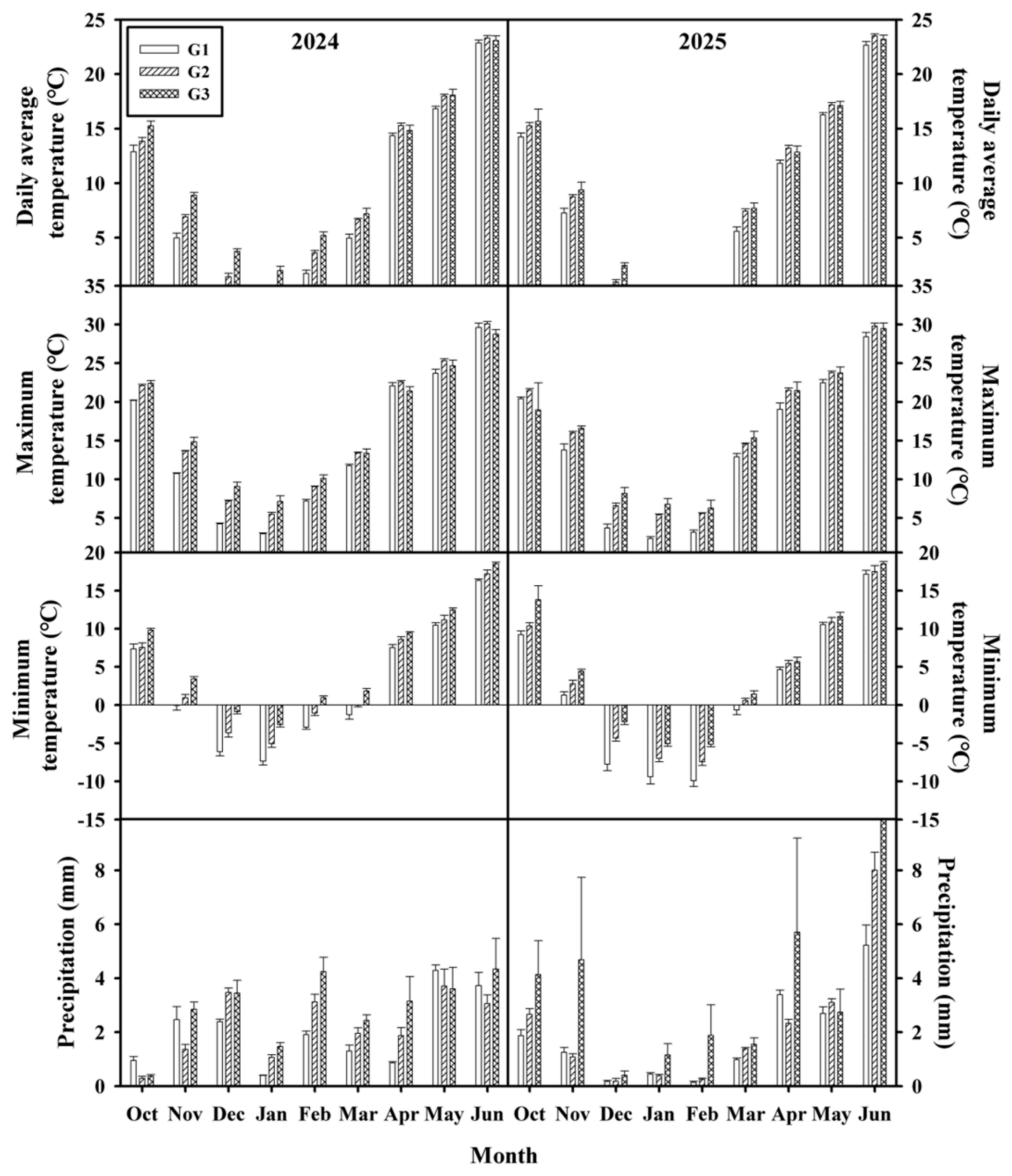
Monthly weather conditions during the growing seasons of wheat by regional groups (G1–G3) in 2024 (**left**) and 2025 (**right**). Values are presented as means ± SE of four locations within each agro-climatic group. Effects of year, month, agro-climatic group, Year × Group, and Month × Group were evaluated using linear mixed-effects models, with detailed statistical results provided in Table S5.

Meteorological data from 2024 and 2025 showed distinct regional patterns in air temperature throughout the overwintering and early growing season period (October–March; Figure 5). Mean and minimum temperatures were higher in G3 than in G1 by up to approximately 2–3 °C. In mid-winter (December–February), minimum temperatures in G1 fell to approximately −10 °C, whereas those in G3 remained relatively high at approximately −3 to −4 °C. G2 exhibited intermediate conditions, with minimum temperatures of approximately −7 to −8 °C. The mixed-effects analysis showed significant effects of year, month, and agro-climatic group on mean, maximum, and minimum temperatures (Table S5). Significant Month × Group interactions were detected for mean and maximum temperatures, indicating that regional temperature differences varied among months. Precipitation varied among months, whereas the effects of year and agro-climatic group and their interactions were not significant.

**Figure 6:**
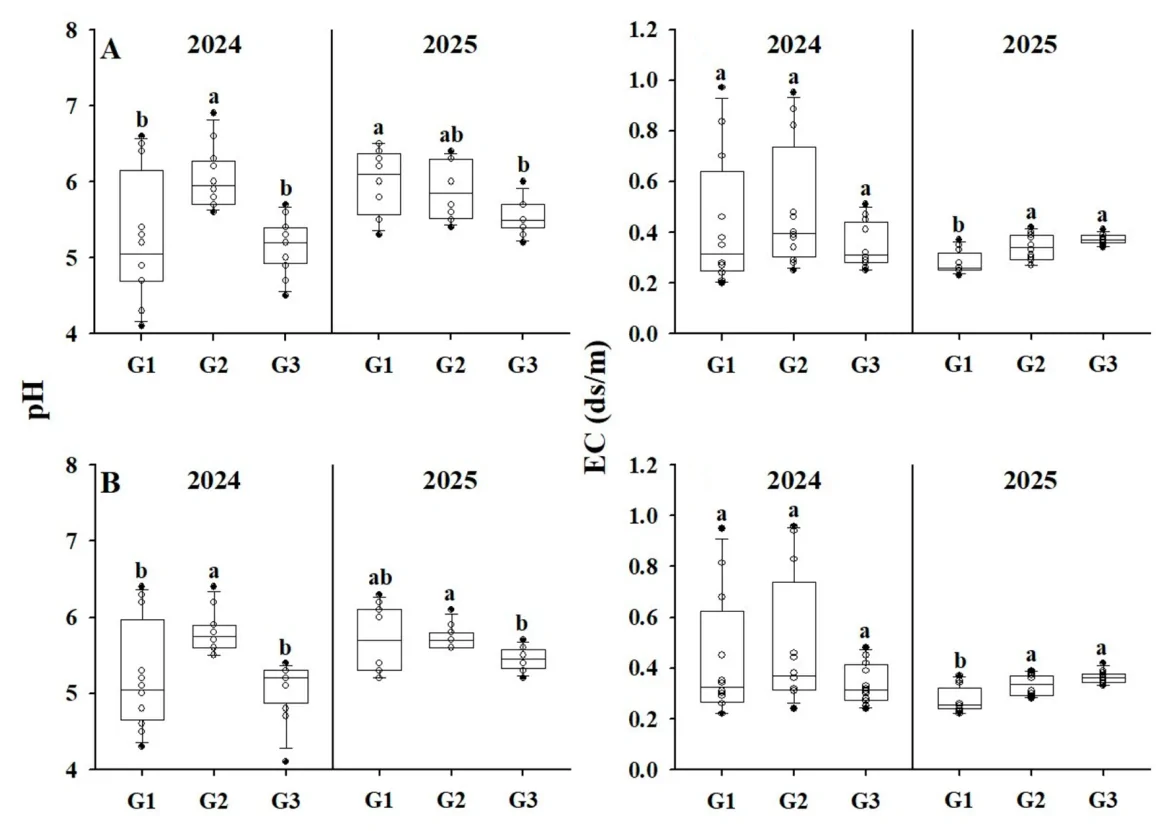
Soil pH and electrical conductivity (EC) of wheat fields among regional groups (G1–G3). Soil samples were collected at the heading (**A**) and harvest (**B**) stages at each experimental site during the 2024 and 2025 growing seasons. Boxplots are presented as described in Figure 2. Individual points represent composite soil samples from replicate sampling plots (*n* = 12 per agro-climatic group in each year). Different letters indicate significant differences among agro-climatic groups within each year based on estimated marginal means with Tukey adjustment (*p* < 0.05). Effects of group, year, and the Group × Year interaction were evaluated using linear mixed-effects models, with detailed statistical results provided in Table S6.

**Figure 7:**
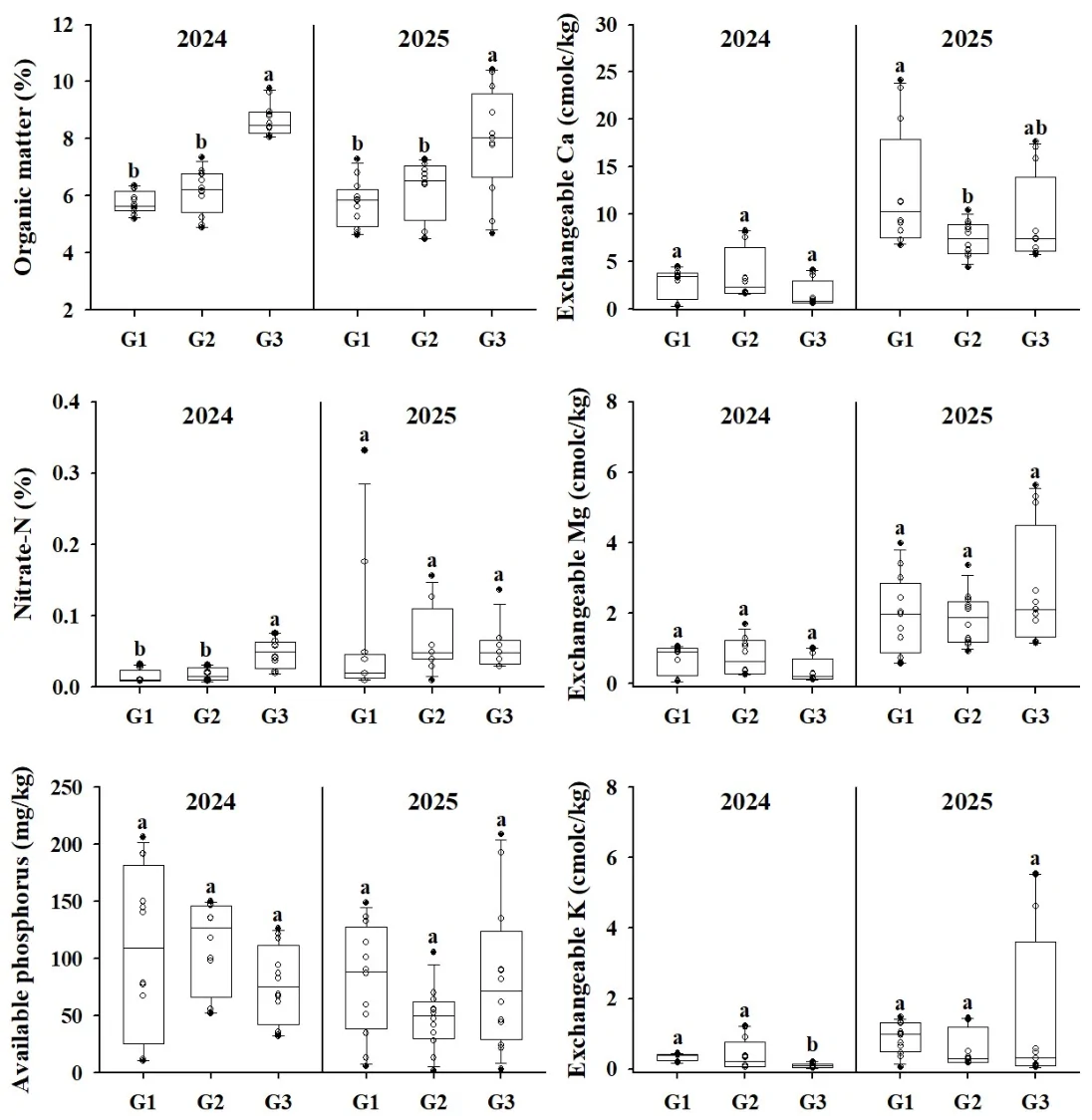
Soil chemical properties of wheat fields among regional groups (G1–G3) at the heading stage in 2024 (**left**) and 2025 (**right**). Boxplots are presented as described in Figure 2. Individual points represent composite soil samples from replicate sampling plots (*n* = 12 per agro-climatic group in each year). Different letters indicate significant differences among agro-climatic groups within each year based on estimated marginal means with Tukey adjustment (*p* < 0.05). Effects of group, year, and the Group × Year interaction were evaluated using linear mixed-effects models, with detailed statistical results provided in Table S7.

Soil properties varied among agro-climatic groups and years (Figure 6 and Figure 7). The mixed-effects analysis showed no significant overall group effects on soil pH or EC, although significant year and Group × Year interaction effects were detected for both variables (Table S6). At the heading stage, soil organic matter differed significantly among agro-climatic groups, whereas nitrate-N showed a significant year effect but no significant overall group effect (Table S7). Available phosphorus and exchangeable Ca, Mg, and K showed significant year and Group × Year interaction effects, although their overall group effects were not significant. These results indicate that regional differences in several soil chemical properties varied between years rather than showing consistent differences among agro-climatic groups.

### 2.5. Relationships of Grain Yield with Environmental Factors and Agronomic Traits

Mixed-effects regression analysis showed that winter minimum temperature was positively associated with grain yield (standardized β = 0.338, *p* = 0.034), whereas soil organic matter, soil NO_3_^−^-N, and available phosphorus were not significantly associated with grain yield (*p* > 0.05; Table S10). Among the major agronomic traits, spike number (β = 0.452, *p* < 0.001), spikelets per spike (β = 0.482, *p* < 0.001), and ripening rate (β = 0.293, *p* = 0.001) were positively associated with grain yield, whereas thousand-grain weight was not significantly associated with grain yield (*p* = 0.089; Table S10).

### 2.6. Quality and Functional Components of Harvested Seeds by Region

**Figure 8:**
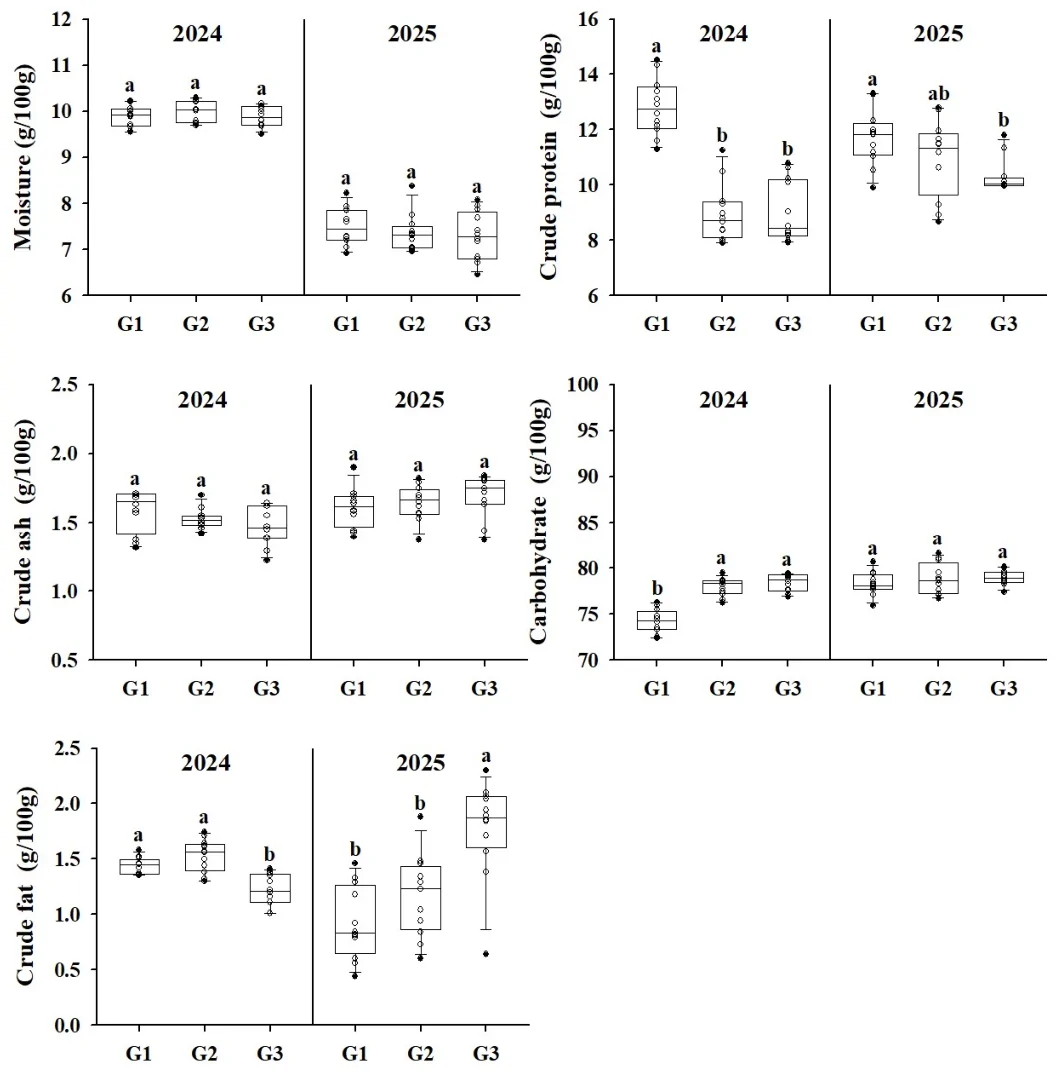
Proximate compositions of wheat grain among regional groups (G1–G3) in 2024 (**left**) and 2025 (**right**). All values are expressed on a dry-weight basis (DW). Boxplots are presented as described in Figure 2. Individual points represent independent grain samples from replicate sampling plots (*n* = 12 per agro-climatic group in each year). Different letters indicate significant differences among agro-climatic groups within each year based on estimated marginal means with Tukey adjustment (*p* < 0.05). Effects of group, year, and the Group × Year interaction were evaluated using linear mixed-effects models, with detailed statistical results provided in Table S8.

Proximate composition analysis showed differences in several grain quality traits among agro-climatic groups and years (Figure 8). Crude protein content was significantly higher in G1 than in G2 and G3 in both 2024 and 2025. Carbohydrate content was significantly higher in G2 and G3 than in G1 in 2024, whereas no significant differences among groups were observed in 2025. Crude fat content showed contrasting regional patterns between years, with lower values in G3 than in G1 and G2 in 2024 but higher values in G3 in 2025. Mixed-effects analysis showed significant overall group effects on crude protein, carbohydrate, and crude fat, with significant Group × Year interactions for all three traits (Table S8). Moisture and crude ash showed no significant overall group effects, although significant year effects were detected for both traits and a significant Group × Year interaction was observed for crude ash.

**Figure 9:**
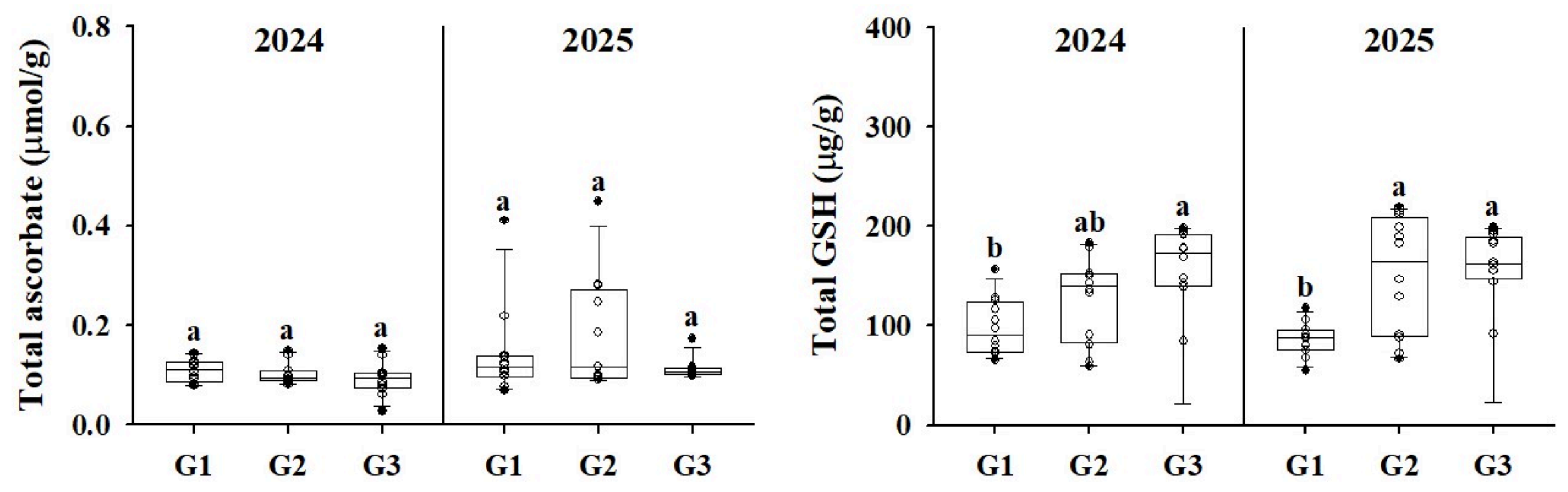
Total ascorbate (AsA) and glutathione (GSH) contents of wheat grain among regional groups (G1–G3) in 2024 (**left**) and 2025 (**right**). All values are expressed on a dry-weight basis (DW). Boxplots are presented as described in Figure 2. Individual points represent independent grain samples from replicate sampling plots (*n* = 12 per agro-climatic group in each year). Different letters indicate significant differences among agro-climatic groups within each year based on estimated marginal means with Tukey adjustment (*p* < 0.05). Effects of group, year, and the Group × Year interaction were evaluated using linear mixed-effects models, with detailed statistical results provided in Table S8.

**Figure 10:**
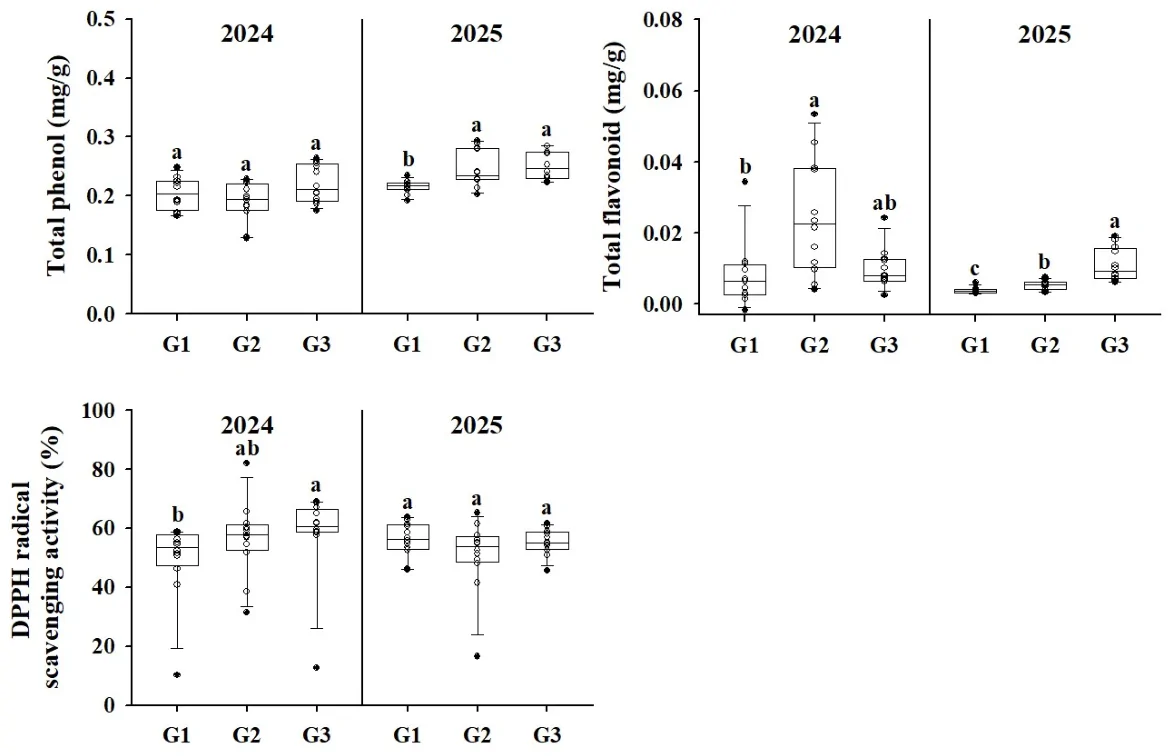
Phenolic and flavonoid contents and DPPH radical-scavenging activity of wheat grain among agro-climatic groups (G1–G3) in 2024 (**left**) and 2025 (**right**). All values are expressed on a dry-weight basis (DW). Boxplots are presented as described in Figure 2. Individual points represent independent grain samples from replicate sampling plots (*n* = 12 per agro-climatic group in each year). Different letters indicate significant differences among agro-climatic groups within each year based on estimated marginal means with Tukey adjustment (*p* < 0.05). Effects of group, year, and the Group × Year interaction were evaluated using linear mixed-effects models, with detailed statistical results provided in Table S8.

Total ascorbate content showed no significant overall group effect, although a significant year effect was detected (Figure 9; Table S8). Total GSH content showed a significant Group × Year interaction, whereas the overall group and year effects were not significant, indicating that regional differences in GSH varied between years. Secondary metabolite traits also showed year-dependent regional patterns (Figure 10). Total flavonoid content was higher in G2 than in G1 in 2024, whereas in 2025 significant differences were observed among the groups in the order G3 > G2 > G1. Total phenol content did not differ significantly among groups in 2024, whereas G2 and G3 showed higher values than G1 in 2025. Significant Group × Year interactions were detected for both total flavonoid and total phenol contents. DPPH radical-scavenging activity showed regional differences only in 2024, whereas the overall effects of group, year, and the Group × Year interaction were not significant.

### 2.7. PCA Based on Regional Quality Characteristics

**Figure 11:**
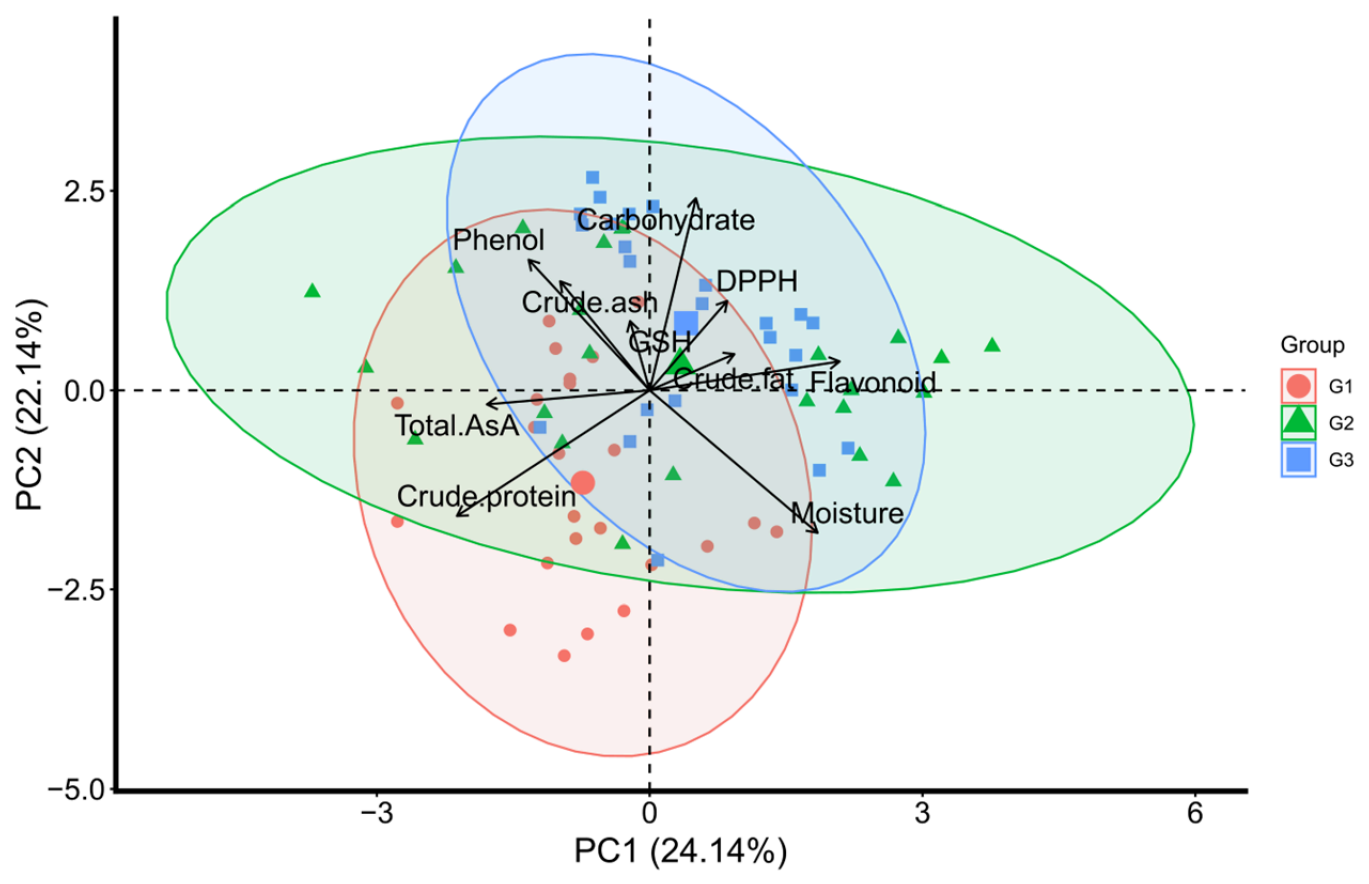
Principal component analysis biplot of wheat quality traits at harvest by regional groups (G1–G3) in 2024 and 2025. The analysis included 24 plot-level observations per agro-climatic group (*n* = 72 in total). Eigenvalues, explained variance, variable loadings, and contributions are provided in Table S9.

PCA was conducted using grain quality and functional component traits (Figure 11; Table S9). The first two principal components explained 46.3% of the total variation, with PC1 and PC2 accounting for 24.1% and 22.1%, respectively. PC1 was mainly associated with crude protein, total flavonoid, moisture, and total ascorbate, whereas PC2 was primarily associated with carbohydrate, moisture, total phenol, and crude protein. The PCA biplot showed differentiation in grain quality and functional component profiles among the three agro-climatic groups. G1 was positioned closer to crude protein and total ascorbate, whereas G2 was more closely associated with total flavonoid and crude fat. G3 was positioned closer to carbohydrate, total GSH, and crude ash. However, considerable overlap among the groups and the 53.7% of variation not explained by PC1 and PC2 indicate that the regional differentiation should be interpreted cautiously.

### 2.8. Integrated Evaluation of Regional Wheat Production Performance

**Figure 12:**
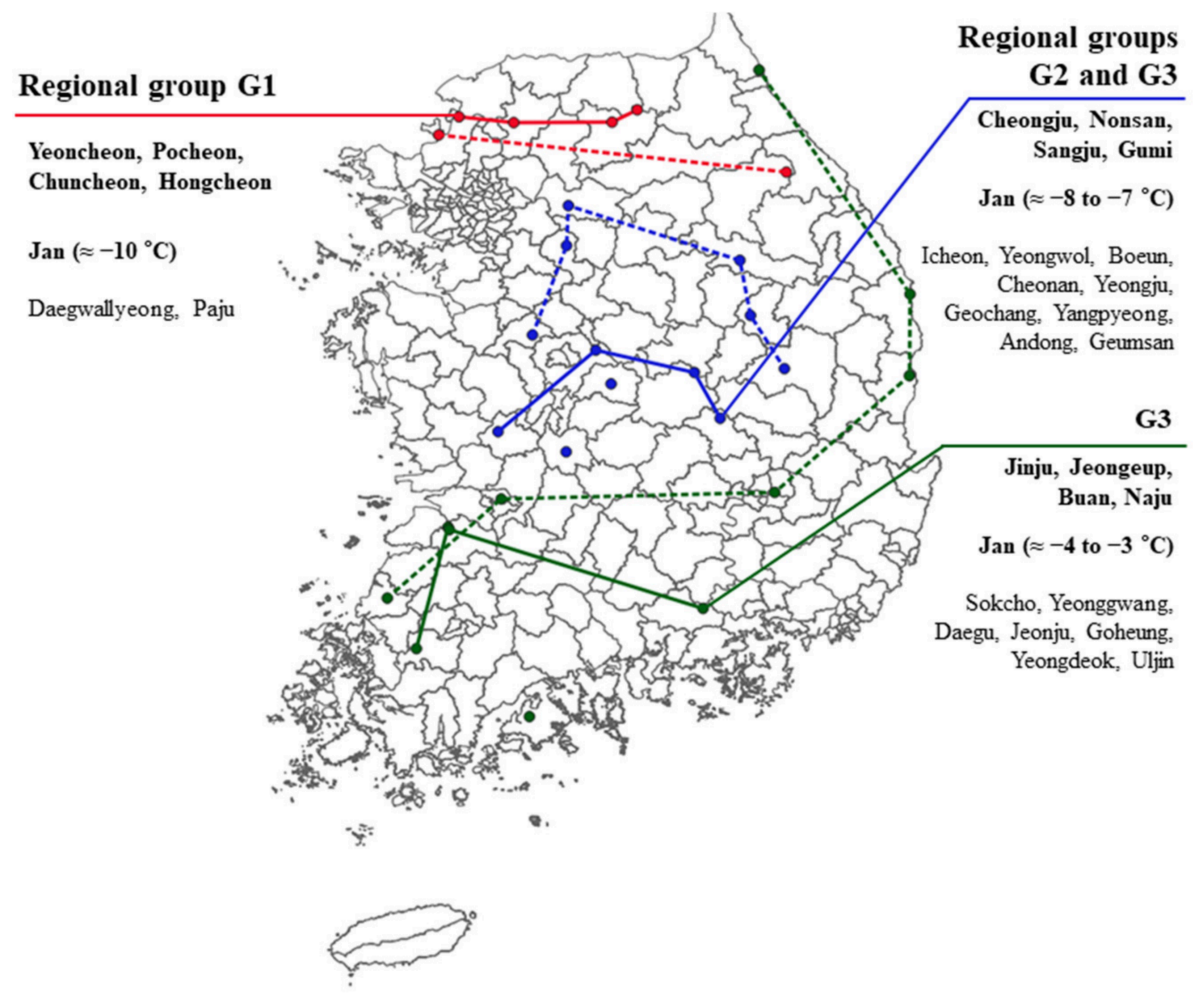
Regional patterns of wheat production performance across the study areas in 2024 and 2025. Dashed lines of the same color indicate areas with the same January mean minimum temperature as the corresponding solid lines and are shown for climatic reference only, not as predicted cultivation boundaries.

The integrated analysis of growth, yield, and grain quality traits further showed that cultivation performance differed considerably among regions (Figure 12). G1 showed relatively poorer growth and yield performance but had higher values for some quality traits, especially crude protein and total phenol contents. In contrast, G2 was intermediate in terms of heading, tiller number, biomass, and yield, with moderate levels of flavonoids and GSH. G3 showed the strongest overall performance, with earlier heading, consistently higher growth and yield components, and higher carbohydrate, crude fat, and antioxidant-related traits.

The regional distribution was consistent with contrasting environmental conditions, as G3 represented sites with milder winter conditions and higher soil organic matter and nitrogen contents than G1, which was associated with cooler temperatures and relatively low-fertility soils. In summary, highly significant differences in cultivation suitability were observed among the regions studied. Overall, G3 showed the highest regional production performance, G2 exhibited intermediate performance, and G1 showed comparatively lower production performance.

## 3. Discussion

### 3.1. Productivity Differences Based on Growth and Yield Characteristics

In South Korea, clear regional differences in wheat growth, yield, and grain quality were observed among the three agro-climatic zones (G1–G3). Although environmental conditions varied between years, G3 generally showed greater growth and yield performance than G1, although the magnitude of regional differences varied between years. The higher productivity of G3 was characterized by earlier heading, greater shoot biomass and generally greater spike numbers. These traits are widely recognized as important agronomic determinants of wheat yield potential [4,14], and their occurrence in G3 was consistent with the higher grain yields observed in this region. This interpretation was further supported by the mixed-effects regression analysis, in which spike number, spikelets per spike, and ripening rate were positively associated with grain yield, whereas thousand-grain weight was not significantly associated with grain yield (Table S10). These responses may reflect more favorable overwintering conditions and enhanced early vegetative growth. However, these mechanisms were not directly evaluated in the present study and require further investigation. In contrast, the lower productivity observed in G1 was associated with prolonged exposure to low winter temperatures and differences in soil nutrient status. Previous studies have shown that these factors can reduce tiller survival, limit canopy establishment, and decrease assimilate production during grain filling, ultimately resulting in lower productivity [6,15,16].

Despite the lower growth and grain yield observed in G1, crude protein content was higher than in the other regions, whereas other functional components showed year-dependent regional patterns. The combination of lower grain yield and higher crude protein content in G1 may suggest a productivity–quality trade-off; however, this interpretation remains hypothetical because the underlying physiological mechanisms were not directly examined. G2 exhibited intermediate characteristics between G1 and G3 and was generally associated with intermediate winter temperature conditions. These findings are consistent with those of previous field-based studies conducted in South Korea, which reported that regional temperature conditions substantially influence barley growth, yield, and grain quality [7]. Similar responses observed in the present study suggest that differences in winter temperature were associated with the regional variation in wheat performance.

### 3.2. Regional Differences in Weather and Soil Factors

Regional differences in wheat phenology and yield were associated with variation in winter temperature. G3 had winter temperatures 2–3 °C higher than those in G1, coinciding with more favorable overwintering and early crop establishment. Furthermore, milder winter temperatures have been reported to improve tiller survival, accelerate spring regrowth, and increase assimilate accumulation before anthesis [9]. In contrast, G1 experienced longer periods with temperatures below −5 °C, which were associated with less favorable overwintering conditions [6]. Although precipitation differed substantially across regions and years, it was not consistently associated with grain yield. Therefore, winter temperature showed a more consistent association with the observed regional differences than precipitation [17,18]. Soil properties also varied among regions and years and were associated with regional variation in wheat performance. Soil organic matter differed significantly among agro-climatic groups, whereas nitrate–nitrogen showed a significant year effect but no significant overall group effect. Because these soil properties were measured at the heading stage, they may reflect not only inherent soil characteristics but also fertilizer management and crop nutrient uptake. Overall, regional differences in wheat growth and yield were associated with variations in winter temperature and soil nutrient status.

The mixed-effects regression analysis further supported the association between winter temperature and grain yield. Winter minimum temperature was positively associated with grain yield after accounting for the selected soil variables (standardized β = 0.338, *p* = 0.034), whereas soil organic matter, soil NO_3_^−^-N, and available phosphorus were not significantly associated with grain yield. These results suggest that, among the environmental factors evaluated, winter temperature showed the clearest quantitative association with grain yield. However, this relationship should be interpreted as an association rather than a causal effect because of the observational nature of the multi-location field study.

### 3.3. Regional Differences in Functional Quality Characteristics

The composition of functional compounds varied among regions and years. G1 was characterized by higher crude protein content, which may be associated with the colder environmental conditions observed in this region [11]. The combination of lower grain yield and higher crude protein content in G1 may suggest a productivity–quality trade-off; however, this interpretation remains hypothetical because the underlying physiological mechanisms were not directly examined. Flavonoid content showed year-dependent regional differences, with higher values in G2 than in G1 in 2024 and in the order G3 > G2 > G1 in 2025. Flavonoids are important bioactive compounds contributing to the functional properties of wheat grain [19]. Carbohydrate content was higher in G2 and G3 than in G1 in 2024, whereas no significant regional differences were observed in 2025. Similarly, GSH content showed a significant Group × Year interaction, whereas DPPH radical-scavenging activity showed no significant overall group, year, or Group × Year effects. GSH plays an important role in antioxidant defense, ROS detoxification, and cellular redox regulation in plants [10,12], whereas DPPH radical-scavenging activity is commonly used as an indicator of antioxidant capacity in wheat grains [20,21]. In summary, these findings suggest that grain quality and functional components exhibited region- and year-dependent variation.

### 3.4. Integrated Trait Relationships Revealed by PCA

The PCA results provided complementary multivariate summaries of the agronomic and grain quality traits. The agronomic PCA explained 80.6% of the total variation in the first two principal components and indicated regional differentiation, with G3 positioned closer to several yield-related traits and G2 showing a broadly similar pattern. These results suggest that regional differences in agronomic performance were reflected in the multivariate distribution of growth and yield traits [13]. In contrast, the first two principal components of the grain-quality PCA explained only 46.3% of the total variation, and considerable overlap was observed among the three agro-climatic groups. Therefore, the grain-quality PCA indicates only partial regional differentiation and should not be interpreted as evidence of clear separation among regions. Conclusions regarding individual grain quality traits were therefore based primarily on the mixed-effects analyses, with PCA used as a complementary approach to visualize multivariate patterns.

### 3.5. Implications for Regional Cultivation Suitability

These results underscore the importance of considering climatic conditions, soil properties, growth, yield, and grain quality when evaluating regional wheat production performance. Although G1 generally showed lower growth and grain yield, its higher crude protein content may suggest a possible trade-off between productivity and grain quality; however, the underlying physiological mechanisms were not directly evaluated in this study. G2 generally showed intermediate agronomic characteristics between G1 and G3, although its performance varied depending on the trait and year. G3 generally exhibited greater growth and grain yield under the conditions evaluated in this study. Overall, regional wheat production performance was associated with variations in winter temperature conditions and soil nutrient status during crop growth. These findings suggest that regional strategies for wheat production should consider not only grain yield but also grain quality traits and environmental conditions.

Several limitations of this study should be acknowledged. First, this study was based on field observations across naturally occurring agro-climatic regions rather than controlled experiments; therefore, the observed regional differences represent associations rather than definitive causal relationships. Because climatic, soil, and management factors varied simultaneously among locations, the independent contribution of individual environmental variables to wheat performance could not be reliably separated in the present study. In addition, soil organic matter and nutrient concentrations were measured at the heading stage after fertilizer application and substantial crop growth. Therefore, these measurements represent soil nutrient status during crop growth rather than baseline soil fertility at crop establishment. Pre-sowing soil nutrient data were not available for all experimental locations and years. Accordingly, regional differences in these soil properties should be interpreted with caution, as they may partly reflect differences in fertilizer management and crop nutrient uptake among locations. Further multi-year and multi-location studies specifically designed to quantify these relationships using correlation or regression approaches are therefore warranted.

In addition, sowing dates varied among locations and years because field management followed local agronomic practices. Because sowing date can affect tillering, heading, biomass accumulation, grain filling, and yield, variation in sowing date may have contributed to some of the observed regional differences. Therefore, the effects of sowing date cannot be fully separated from those of other environmental factors in the present field-based study. Future studies using standardized sowing dates or explicitly incorporating sowing date into statistical models are needed to better distinguish its contribution from other environmental factors. Finally, this study evaluated only a single wheat cultivar, ‘Saegumgang’, although it is widely cultivated in South Korea. Therefore, the observed regional responses may differ among other wheat cultivars because of cultivar × environment interactions. Future studies incorporating controlled experiments, multivariate modeling, and multiple wheat cultivars are needed to better quantify the relative contributions of individual environmental factors and improve the generalizability of these findings.

## 4. Materials and Methods

### 4.1. Study Regions and Experimental Design

**Figure 13:**
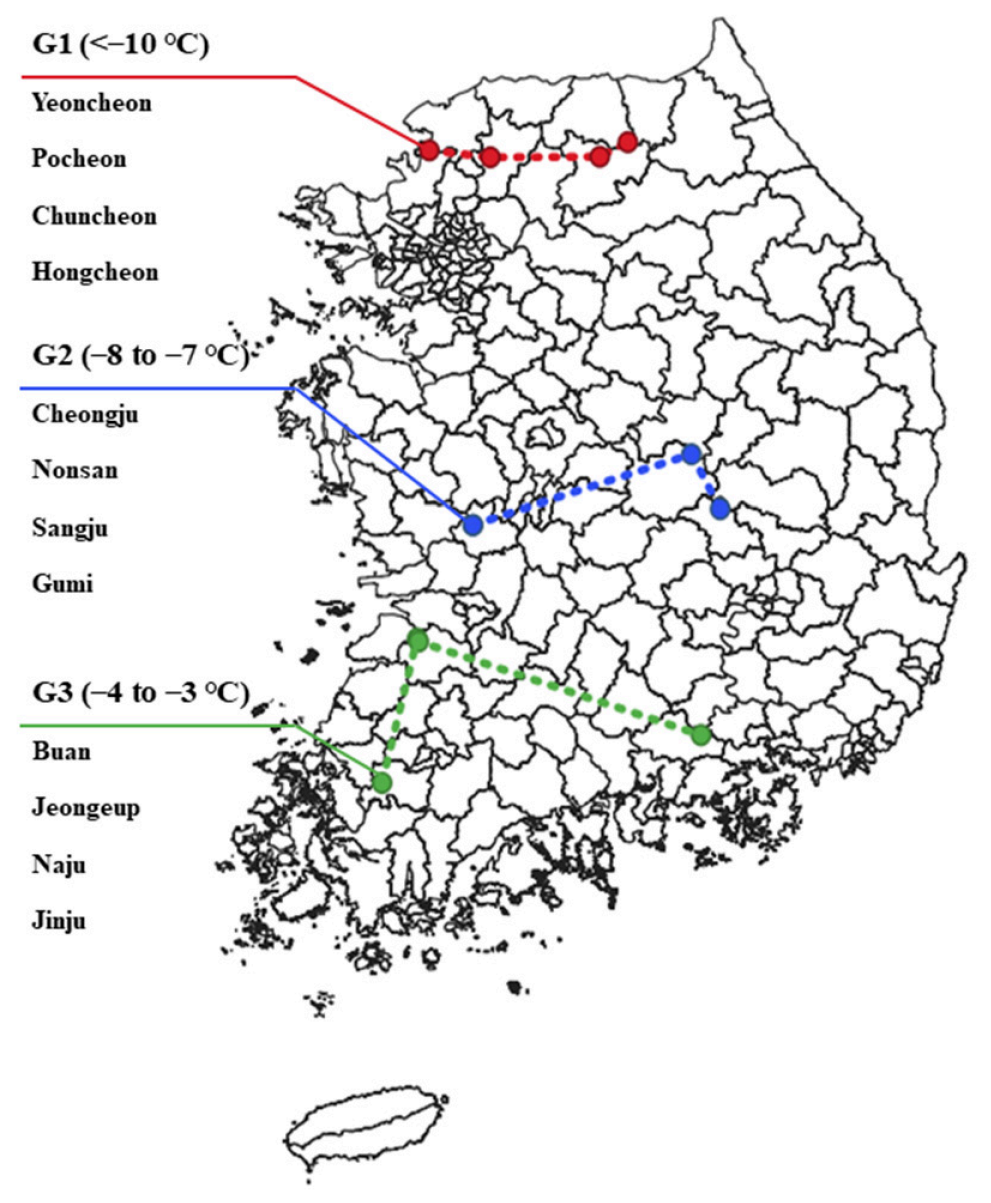
Locations of the 12 wheat field experimental sites classified into three agro-climatic groups (G1–G3) based primarily on January minimum temperature.

Field experiments were conducted during the 2024 and 2025 growing seasons in the major wheat-producing regions of South Korea. Regional classification was primarily based on January minimum temperature. The experimental sites were grouped into three agro-climatic regions (G1–G3), representing contrasting winter temperature conditions (Figure 13). G1 consisted of central inland and high-altitude regions with an average January minimum temperature below −10 °C over the previous five years: Yeoncheon, Pocheon, Chuncheon, and Hongcheon. G2 represented southern inland and plain regions with average January minimum temperatures ranging from −8 to −7 °C: Cheongju, Nonsan, Sangju, and Gumi. G3 represented southern coastal and warmer regions with average January minimum temperatures ranging from −4 to −3 °C: Buan, Jeongeup, Naju, and Jinju.

These classifications were used to represent contrasting winter temperature environments and evaluate their associations with wheat growth, yield, and grain quality. Each agro-climatic group consisted of four geographically independent cultivation locations. At each location, three replicate sampling plots were established within a commercial farmer-managed field and were used as within-location sampling units. Because the study was conducted across naturally occurring agroclimatic regions rather than experimentally manipulated environments, the observed regional differences should be interpreted as associations among multiple environmental factors rather than as direct causal effects of individual variables. No spatial interpolation or predictive suitability modeling was performed. Figure 12 was prepared as an indicative summary of regional wheat production patterns based on the agro-climatic classification of the 12 experimental locations and was not intended to define or predict geographical cultivation boundaries.

### 4.2. Crop Management and Field Measurements

The sowing dates, harvest dates, and field areas of the experimental sites are summarized in Table 1. All experiments were conducted in commercial farmer-managed fields. All experimental sites were planted with ‘Saegumgang’, a widely grown Korean wheat cultivar characterized by high yield and cultivation stability and primarily used for noodle production. Sowing was performed according to local agronomic practices between late October and mid-November, depending on the site. Broadcast sowing was performed at a seeding rate of 150–200 kg ha^−1^. Pest control and weed management were uniformly implemented across all locations following the standard recommendations of the Rural Development Administration [22]. Specifically, the standard N–P_2_O_5_–K_2_O fertilization rate was applied at 91–74–39 kg ha^−1^, respectively, with nitrogen split-applied as basal fertilizer (40%) and topdressing at the spring regrowth stage (60%).

Growth traits were measured at four main growth stages: pre-wintering (9–19 December), post-wintering (13 February–5 March), heading (16 April–13 May), and harvest (30 May–24 June). The measurements included plant height, number of tillers, chlorophyll content (SPAD; SPAD-502, Minolta, Japan), and aboveground biomass. Pre- and post-wintering growth measurements were conducted only during the 2025 growing season, whereas measurements from the heading stage onward, including yield components, grain quality, meteorological conditions, and soil properties, were performed in both 2024 and 2025 growing seasons. Yield components, including stem length, spike length, spike number, spikelets per spike, thousand-grain weight, ripening rate, and grain yield, were determined at harvest. Grain yield was determined from three sampling plots (4 m × 5 m) established within each commercial farmer-managed field and expressed on a hectare basis after adjustment to 14% grain moisture.

### 4.3. Weather Data Collection and Analysis

The meteorological data used were obtained from the Korea Meteorological Administration [5] and covered the wheat growing period (October–June). Data were obtained from the nearest meteorological station to each experimental site. The monthly mean temperature, minimum temperature, and precipitation were used to characterize the regional climatic conditions during the crop growth period.

### 4.4. Soil Sampling and Analysis

Soil samples were collected from the topsoil layer (0–20 cm) at each site to evaluate soil fertility status. At each location, soil samples were collected from three replicate sampling plots. Within each replicate plot, three subsamples, approximately 300 g each, were randomly collected and combined to form one composite sample. Thus, three independent composite soil samples, corresponding to the three replicate sampling plots, were analyzed at each location. Soil pH and electrical conductivity (EC) were determined at different developmental stages: pre-wintering, post-wintering, heading, and harvest.

At the heading stage, additional analyses were conducted for organic matter, nitrate nitrogen (NO_3_^−^–N), available phosphate (P_2_O_5_), and exchangeable cations (K, Ca, and Mg). Available phosphorus was determined using the Lancaster method and expressed as mg P_2_O_5_ kg^−1^. Exchangeable K, Ca, and Mg were extracted with 1 N ammonium acetate and quantified by atomic absorption spectroscopy, with the results expressed as cmolc kg^−1^. All analyses were performed according to the standard procedures described in the Soil and Plant Analysis Manual [22].

### 4.5. Analysis of Grain Composition and Functional Components

Grain samples (1000 g per replicate) were collected separately from each of the three replicate sampling plots (4 m × 5 m) at harvest, resulting in three independent grain samples per field. The samples were dried and ground before analysis. Proximate composition, including the contents of crude protein, carbohydrate, crude fat, moisture, and ash, was determined following the methods described by [23]. Crude protein was analyzed using the micro-Kjeldahl method, ash content was determined by incineration at 550 °C, and crude fat content was measured using the Soxhlet extraction method. Total carbohydrate content was calculated by difference [23] as follows: carbohydrate (%) = 100 − [moisture (%) + crude protein (%) + crude fat (%) + crude ash (%)].

Functional components were evaluated by measuring total ascorbate and GSH contents. Total ascorbate analysis involved suspending 0.1 g of ground grain in 5% metaphosphoric acid, centrifuging, and measuring the absorbance of the supernatant at 525 nm, using ascorbic acid as a standard [24]. Total glutathione content was determined similarly, with absorbance measured at 412 nm for 1 min using glutathione as a standard [25]. Total phenolic content, total flavonoid content, and DPPH radical-scavenging activity were determined using previously established methods [26]. Briefly, to evaluate these activities, 0.5 g of the dried and ground grain samples was mixed with 10 mL of 99.9% ethanol and agitated at 120 rpm for 24 h at 27 °C in a shaking water bath. The mixture was centrifuged at 13,000× *g* for 10 min using a high-speed refrigerated centrifuge (VS-24SMTI, Vision Scientific Co., Ltd., Daejeon, Republic of Korea), and the resulting supernatant was collected for subsequent assays. All other analytical procedures strictly followed the protocols described in the aforementioned studies. The three independently collected grain samples from the replicate sampling plots were treated as biological replicates for grain composition and functional component analyses.

### 4.6. Statistical Analysis

Growth, yield, soil, and grain quality data were analyzed using a linear mixed-effects model to account for the hierarchical structure of the study. Agro-climatic group (G1–G3), year, and the Group × Year interaction were treated as fixed effects, whereas location nested within group and replicate sampling plot nested within-location were treated as random effects. The significance of the group, year, and Group × Year interaction effects was evaluated at *p* < 0.05. Pairwise comparisons among agro-climatic groups within each year were performed using estimated marginal means with Tukey adjustment at *p* < 0.05. To quantify the relationships between grain yield and selected environmental factors and major agronomic traits, additional linear mixed-effects regression analyses were performed. Grain yield and all continuous predictors were standardized to z-scores prior to analysis. Winter minimum temperature (December–February), soil organic matter, soil NO_3_^−^-N, and available phosphorus were simultaneously included in a multivariable mixed-effects model. Major agronomic traits were evaluated in separate mixed-effects models because of relatively strong correlations among traits. Year was included as a fixed effect, and location nested within agro-climatic group was included as a random effect. The resulting standardized regression coefficients (β) were used to quantify the strength and direction of the associations. Two separate PCAs were conducted: one using agronomic and yield-related traits and the other using grain quality and functional component traits. PCA was performed using plot-level observations from the three replicate sampling plots at each of the four locations within each agro-climatic group. Data from 2024 and 2025 were combined, resulting in 24 observations per group (12 observations per group per year) and 72 observations in total for each PCA. Before PCA, all variables were standardized to a mean of zero and unit variance. Eigenvalues, variable loadings, and variable contributions for the agronomic and grain-quality PCAs are provided in Supplementary Tables S4 and S9, respectively. Statistical analyses were performed using R software (version 4.2.1) and XLSTAT (2023).

## 5. Conclusions

This study evaluated regional differences in wheat growth, yield, grain quality, climatic conditions, and soil properties across three agro-climatic zones (G1–G3) in South Korea. Under the conditions evaluated in this study, wheat grown in the warmer southern coastal region (G3) generally exhibited higher growth and grain yield, whereas wheat grown in the colder inland region (G1) had higher crude protein content, suggesting a possible regional trade-off between productivity and grain quality. Among the environmental factors evaluated, winter minimum temperature showed a significant positive association with grain yield, whereas the evaluated soil nutrient variables were not significantly associated with grain yield. However, because the present study was based on four locations per agro-climatic group, two growing seasons, and a single wheat cultivar (‘Saegumgang’), these findings should be considered preliminary regional evidence rather than definitive criteria for cultivation suitability. Overall, this study provides field-based evidence of regional variation in wheat production performance and may support the development of region-specific cultivation strategies that consider both productivity and grain quality under changing climatic conditions. Further studies across additional years, locations, and wheat cultivars are needed to validate these regional patterns and improve the generalizability of the findings.

## Figures and Tables

**Table 1:** Sowing dates, harvest dates, and field areas of the 12 experimental locations in 2024 and 2025.

Group	Location	Sowing Date (MM.DD)		Field Area (ha)		Harvest Date (MM.DD)	
		2024	2025	2024	2025	2024	2025
G1	Yeoncheon	11.03	11.14	0.7	0.3	6.18	6.23
	Pocheon	11.05	11.04	0.5	0.1	6.15	6.24
	Chuncheon	10.24	10.20	1.0	0.4	6.13	6.14
	Hongcheon	10.23	10.24	0.1	0.1	6.13	6.14
G2	Cheongju	10.29	11.10	0.1	0.4	6.16	6.24
	Nonsan	10.25	11.05	1.0	1.5	6.10	6.11
	Sangju	11.03	11.11	0.5	0.3	6.07	6.13
	Gumi	10.27	10.23	0.3	0.3	6.09	6.13
G3	Jinju	10.30	11.02	1.5	0.4	6.03	6.09
	Jeongeup	10.27	11.10	0.5	0.4	6.02	6.10
	Buan	10.25	10.30	0.1	0.4	6.02	6.10
	Naju	10.26	11.02	0.9	0.4	5.30	6.09

## Data Availability

Data is contained within the article. The data presented in this study are available upon request from the corresponding author.

## Supplementary Materials

The following supporting information can be downloaded at: https://www.mdpi.com/article/10.3390/plants15182852/s1, Table S1. *p*-values for the effects of agro-climatic group on pre- and post-overwintering growth traits of wheat in 2025. Table S2. Fixed-effect *p*-values from linear mixed-effects models for heading-stage growth traits of wheat. Table S3. Fixed-effect *p*-values from linear mixed-effects models for harvest-stage growth and yield-related traits of wheat. Table S4. Eigenvalues, explained variance, variable loadings, and contributions for the PCA of agronomic and yield-related traits. Table S5. Fixed-effect *p*-values from linear mixed-effects models for meteorological variables. Table S6. Fixed-effect *p*-values from linear mixed-effects models for soil pH and electrical conductivity (EC). Table S7. Fixed-effect *p*-values from linear mixed-effects models for soil chemical properties measured at the heading stage. Table S8. Fixed-effect *p*-values from linear mixed-effects models for wheat grain quality and functional components. Table S9. Eigenvalues, explained variance, variable loadings, and contributions for the PCA of grain quality and functional component traits. Table S10. Mixed-effects regression analysis of grain yield in relation to selected environmental factors and major agronomic traits.
