# Supplementary material for: Agro-Climatic Variation in Wheat Growth, Yield, and Grain Quality Across South Korea
Source: Plants (Basel). 2026 Sep 18;15(18):2852. doi: 10.3390/plants15182852 (PMC13610783; doi:10.3390/plants15182852)
Supplement: Supplementary file 1 [file plants-15-02852-s001.zip › plants-4542430-supplementary.pdf]

Table S1. P-values for the effects of agro-climatic group on pre- and post-overwintering growth traits of wheat in 2025.

Trait	Group	
	Pre-overwintering	Post-overwintering
Plant height	ns	ns
Tiller number	ns	ns

ns, not significant (p ≥ 0.05).

Table S2. Fixed-effect P-values from linear mixed-effects models for heading-stage growth traits of wheat.

Trait	Group	Year	Group x Year
Plant height	ns	< 0.001	< 0.01
Tiller number	< 0.05	< 0.01	< 0.001
SPAD value	ns	ns	< 0.001
Shoot dry weight	< 0.001	< 0.001	< 0.001
Heading date	< 0.05	< 0.001	< 0.001

ns, not significant (p ≥ 0.05).

Table S3. Fixed-effect P-values from linear mixed-effects models for harvest-stage growth and yield-related traits of wheat.

Trait	Group	Year	Group x Year
Stem length	< 0.05	< 0.01	< 0.001
Spike length	< 0.001	ns	< 0.001
Spike number	< 0.01	< 0.001	< 0.001
Spikelets per spike	< 0.001	< 0.001	< 0.001
Ripened grain (%)	< 0.001	ns	< 0.001
Thousand-grain weight	ns	ns	< 0.001
Grain yield	< 0.001	ns	< 0.01

ns, not significant (p ≥ 0.05).

Table S4. Eigenvalues, explained variance, variable loadings, and contributions for the PCA of agronomic and yield-related traits.

(A) Eigenvalues and explained variance

Component	Eigenvalue	Explained variance (%)	Cumulative variance (%)
PC1	4.83	69.0	69.0
PC2	0.81	11.6	80.6

(B) Variable loadings and contributions

Variable	PC1 loading	PC1 contribution (%)	PC2 loading	PC2 contribution (%)
Stem length	0.192	3.7	0.589	34.7
Spike length	-0.418	17.5	-0.154	2.4
Spike number	-0.457	20.9	0.385	14.9
Spikelets per spike	-0.475	22.5	-0.291	8.5
Ripened grain (%)	-0.375	14.0	0.137	1.9
Thousand-grain weight	0.107	1.1	0.524	27.5
Grain yield	-0.449	20.2	0.321	10.3

PC, principal component. PCA was performed using standardized plot-level observations from 2024 and 2025 (n = 72).

Table S5. Fixed-effect P-values from linear mixed-effects models for meteorological variables.

Trait	Year	Month	Group	Year x Group	Month x Group
Daily average temperature	< 0.001	< 0.001	< 0.01	ns	< 0.001
Maximum temperature	< 0.01	< 0.001	< 0.01	ns	< 0.01
Minimum temperature	< 0.001	< 0.001	< 0.001	ns	ns
Precipitation	ns	< 0.001	ns	ns	ns

ns, not significant ($p \geq 0.05$).

Table S6. Fixed-effect P-values from linear mixed-effects models for soil pH and electrical conductivity (EC).

Trait	Group	Year	Group x Year
pH A	ns	< 0.001	< 0.001
pH B	ns	< 0.001	< 0.01
EC A	ns	< 0.01	< 0.05
EC B	ns	< 0.01	< 0.05

A and B indicate measurements at the heading and harvest stages, respectively. ns, not significant ($p \geq 0.05$).

Table S7. Fixed-effect P-values from linear mixed-effects models for soil chemical properties measured at the heading stage.

Trait	Group	Year	Group x Year
Organic matter	< 0.01	ns	ns
Nitrate-N	ns	< 0.05	ns
Available phosphorus	ns	< 0.01	< 0.05
Exchangeable Ca	ns	< 0.001	< 0.01
Exchangeable Mg	ns	< 0.001	< 0.01
Exchangeable K	ns	< 0.001	< 0.05

ns, not significant ($p \geq 0.05$).

Table S8. Fixed-effect P-values from linear mixed-effects models for wheat grain quality and functional components.

Trait	Group	Year	Group x Year
Moisture	ns	< 0.001	ns
Crude ash	ns	< 0.001	< 0.01
Crude fat	< 0.05	ns	< 0.001
Crude protein	< 0.01	< 0.001	< 0.001
Carbohydrate	< 0.01	< 0.001	< 0.001
Total ascorbate	ns	< 0.01	ns
Total GSH	ns	ns	< 0.001
Total phenol	ns	< 0.001	< 0.01
Total flavonoid	ns	< 0.001	< 0.001
DPPH radical scavenging activity	ns	ns	ns

ns, not significant ($p \geq 0.05$).

Table S9. Eigenvalues, explained variance, variable loadings, and contributions for the PCA of grain quality and functional component traits.

(A) Eigenvalues and explained variance

Component	Eigenvalue	Explained variance (%)	Cumulative variance (%)
PC1	2.41	24.1	24.1
PC2	2.21	22.1	46.3

(B) Variable loadings and contributions

Variable	PC1 loading	PC1 contribution (%)	PC2 loading	PC2 contribution (%)
Moisture	0.412	17.0	-0.417	17.4
Crude ash	-0.219	4.8	0.319	10.2
Crude fat	0.208	4.3	0.106	1.1
Crude protein	-0.473	22.4	-0.368	13.5
Carbohydrate	0.113	1.3	0.560	31.4
Total ascorbate	-0.399	15.9	-0.041	0.2
Total GSH	-0.048	0.2	0.203	4.1
Total phenol	-0.297	8.8	0.382	14.6
Total flavonoid	0.466	21.7	0.085	0.7
DPPH radical scavenging activity	0.190	3.6	0.261	6.8

PC, principal component. PCA was performed using standardized plot-level observations from 2024 and 2025 (n = 72).

Table S10. Mixed-effects regression analysis of grain yield in relation to selected environmental factors and major agronomic traits.

(A) Environmental factors

Predictor	Standardized β	SE	df	t-value	*P*-value
Winter minimum temperature (Dec–Feb)	0.338	0.156	60.28	2.166	0.034
Soil organic matter	−0.003	0.130	65.94	−0.025	0.980
Soil NO_3^--N	−0.033	0.065	57.68	−0.509	0.613
Available phosphorus	0.061	0.066	56.82	0.927	0.358

(B) Major agronomic traits

Predictor	Standardized β	SE	df	t-value	*P*-value
Spike number	0.452	0.092	67.75	4.921	< 0.001
Spikelets per spike	0.482	0.083	68.95	5.838	< 0.001
Ripening rate	0.293	0.086	67.76	3.414	0.001
Thousand-grain weight	0.132	0.076	63.01	1.730	0.089

Note: Continuous variables, including grain yield and predictors, were standardized to z-scores prior to analysis; thus, β represents the standardized regression coefficient. Environmental factors were simultaneously included in a multivariable linear mixed-effects model, whereas major agronomic traits were evaluated in separate mixed-effects models because of relatively strong correlations among traits. Year was included as a fixed effect, and location nested within agro-climatic group was included as a random effect. Soil variables represent soil nutrient status measured at the heading stage.
